# A systematic review of shared decision making interventions in chronic conditions: a review protocol

**DOI:** 10.1186/2046-4053-3-38

**Published:** 2014-04-15

**Authors:** Michael R Gionfriddo, Aaron L Leppin, Juan P Brito, Annie LeBlanc, Kasey R Boehmer, Megan A Morris, Patricia J Erwin, Larry J Prokop, Claudia L Zeballos-Palacios, German Malaga, J Jaime Miranda, Heidi M McLeod, René Rodríguez-Gutiérrez, Rongchong Huang, Oscar L Morey-Vargas, Mohammad Hassan Murad, Victor M Montori

**Affiliations:** 1 Knowledge and Evaluation Research Unit, Mayo Clinic, 200 First Street SW, Rochester, MN, USA; 2 Mayo Graduate School, Mayo Clinic, 200 First Street SW, Rochester, MN, USA; 3 Division of Endocrinology, Diabetes, Metabolism, and Nutrition, Mayo Clinic, 200 First Street SW, Rochester, MN 55905, USA; 4 Division of Health Care Policy and Research, Mayo Clinic, 200 First Street SW, Rochester, MN, USA; 5 Mayo Clinic Libraries, Mayo Clinic, Rochester, MN, USA; 6 Unidad de Conocimiento y Evidencia (CONEVID), Universidad Peruana Cayetano Heredia, Lima, Peru; 7 Department of Medicine, School of Medicine, Universidad Peruana Cayetano Heredia, Lima, Peru; 8 CRONICAS Center of Excellence in Chronic Diseases, Universidad Peruana Cayetano Heredia, Lima, Peru; 9 Endocrinology Division, University Hospital ‘Dr. José E Gonzalez’, Universidad Autónoma de Nuevo León, Monterrey, Mexico; 10 Department of Cardiology, the First Affiliated Hospital of Dalian Medical University, Dalian 116011, China; 11 Division of Preventive, Occupational and Aerospace Medicine, Mayo Clinic, 200 First Street SW, Rochester, MN, USA

**Keywords:** Protocol, Systematic review, Chronic condition, Chronic disease, Chronic illness, Shared decision making, Decision making, Decision support tool, Decision aid

## Abstract

**Background:**

Chronic conditions are a major source of morbidity, mortality and cost worldwide. Shared decision making is one way to improve care for patients with chronic conditions. Although it has been widely studied, the effect of shared decision making in the context of chronic conditions is unknown.

**Methods/Design:**

We will perform a systematic review with the objective of determining the effectiveness of shared decision making interventions for persons diagnosed with chronic conditions. We will search the following databases for relevant articles: PubMed, Scopus, Ovid MEDLINE, Ovid EMBASE, Ovid EBM Reviews CENTRAL, CINAHL, and Ovid PsycInfo. We will also search clinical trial registries and contact experts in the field to identify additional studies. We will include randomized controlled trials studying shared decision making interventions in patients with chronic conditions who are facing an actual decision. Shared decision making interventions will be defined as any intervention aiming to facilitate or improve patient and/or clinician engagement in a decision making process. We will describe all studies and assess their quality. After adjusting for missing data, we will analyze the effect of shared decision making interventions on outcomes in chronic conditions overall and stratified by condition. We will evaluate outcomes according to an importance ranking informed by a variety of stakeholders. We will perform several exploratory analyses including the effect of author contact on the estimates of effect.

**Discussion:**

We anticipate that this systematic review may have some limitations such as heterogeneity and imprecision; however, the results will contribute to improving the quality of care for individuals with chronic conditions and facilitate a process that allows decision making that is most consistent with their own values and preferences.

**Trial registration:**

PROSPERO Registration Number: CRD42013005784

## Background

Nearly half of all Americans have at least one chronic condition [1]. Consequently, chronic conditions account for 78% of all healthcare costs [2] and are responsible for 70% of the deaths that occur in America every year [1]. Worldwide, chronic conditions, such as diabetes, cardiovascular disease, and cancer, are responsible for over 36 million deaths every year [3] and a cost of up to US$23 trillion [4]. In addition to the impact that chronic conditions have at the public health level, they also impose morbidity at the individual patient level. This manifests as increased suffering and diminished quality of life for the patient [5], heightened burden on their caregivers [6-8], and decreased satisfaction from the perspective of the clinician [6]. In the complex and often frustrating context of chronic disease management, approaches to improve care are needed. An increasingly common approach towards this goal is patient engagement. Patient engagement can occur at a variety of levels (for example, encounter, organization, policy) and can vary in its intensity [9]. Shared decision making (SDM) is an example of an approach to patient engagement that occurs at the level of the clinical encounter.

SDM is an approach to clinical encounters that desires the intentional and cooperative involvement of both patients and clinicians in the process of deliberation about care. The goal of this approach is that clinicians and patients will share their knowledge, values, and preferences and deliberate together. Thus, SDM can result in decisions that are more congruent with patient values, preferences, and context, which in turn increase quality of life and improve the likelihood of achieving health goals. SDM could improve clinical outcomes when it leads to decisions patients are more likely to implement and enact over time. SDM may also play a role in improving clinician satisfaction in the care of patients with chronic conditions through facilitation of a stronger clinician-patient relationship and a shared understanding of the treatment goals [10,11].

Multiple studies have shown that SDM interventions can increase patient knowledge, reduce aspects of decisional conflict, the proportion of patients remaining undecided, and the proportion of patients who play a passive role in the decision making process [12-15]. Primarily, these effects have been shown in studies of decision aids. Decision aids are tools which can be used to facilitate SDM and are defined by the International Patient Decision Aids Standards (IPDAS) collaboration as:

‘tools designed to help people participate in decision making about two or more health care options…(they) provide information about the options and help patients to clarify and communicate the personal values they associate with different features of the options’ [16].

A diverse array of patient and clinician training programs has also been developed to implement SDM [17]. However, despite widespread policy interest [18,19] in and research [12-15] around ways to implement SDM, it is unclear what effect SDM has in the context of chronic conditions. To date, no systematic reviews exist on the effect of SDM on outcomes in patients with chronic conditions.

SDM in chronic conditions is different from SDM in acute settings or in the context of one-time decisions because of the ability to revisit and revise the decision. A model of SDM in chronic conditions reflecting this has been proposed [20]. We believe that despite this difference (that is the ability to revisit and revise the decision), SDM in chronic conditions should have similar effects as those seen in systematic reviews that do not discriminate between SDM in chronic and non-chronic conditions (for example, increase knowledge, reduce decisional conflict, reduce proportion undecided, and reduce proportion of patients playing a passive role in decision making); however, it is unknown whether this is the case.

To fill this gap, this study aims to determine the effectiveness of SDM interventions compared to usual care or alternative interventions (for example, patient education or modified intervention) for persons diagnosed with chronic conditions.

## Methods/Design

### Study design

We will conduct a systematic review adhering to the reporting guidelines of the Preferred Reporting Items for Systematic Reviews and Meta-Analyses (PRISMA) statement [21].

### Study registration

This systematic review is registered with PROSPERO (registration number CRD42013005784; http://www.crd.york.ac.uk/PROSPERO).

### Criteria for considering studies for this review

#### Type of studies

We will include randomized controlled trials that compare a SDM intervention to usual care, one or more alternative interventions (for example, patient education or modified intervention), or a combination of usual care and alternative intervention(s).

#### Type of participants

We will include studies involving patients with a diagnosis of one or more chronic conditions, as defined in the US Department of Health and Human Services Strategic Framework for Multiple Chronic Conditions as ‘conditions that last a year or more and require ongoing medical attention and/or limit activities of daily living’ (for example, diabetes, osteoarthritis, substance abuse disorders) [22]. Study eligibility will not be restricted based on age. Patients in eligible studies will need to be facing an actual decision (that is studies will not be eligible if the decision to be made is hypothetical, for example, when patients are asked to make a choice about a decision that will not actually be implemented) and that decision must be able to revisited and revised. In cases where the patient may not have agency to make an informed decision (for example, young pediatrics/seniors with advanced dementia), the caregiver will be considered the decision maker.

#### Type of interventions

We will include studies that evaluate any intervention aiming to improve SDM between clinicians and patients. The concept of SDM has evolved over the past thirty years, from a concept focused on informed consent [23] to one focused on information exchange between clinicians and patients that encompasses not only the risks and benefits of treatment options, but also the patient’s and clinician’s values and preferences [24]. For this review, we used a more current definition of SDM and define a SDM intervention as any intervention that aims to inform patients of the available options and their risks and benefits, and engage patients in a decision making process with their clinician.

#### Type of outcome measures

**Figure 1:**
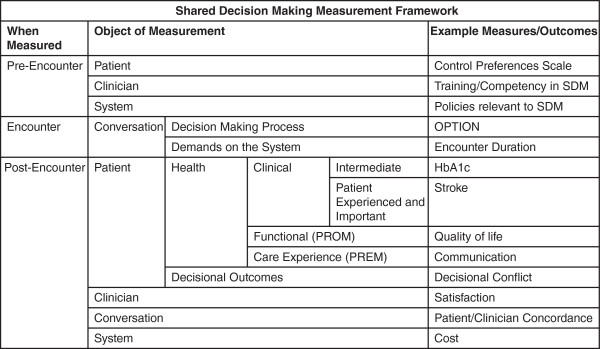
Shared decision making measurement framework.

We will extract all reported outcomes from eligible studies, regardless of the type of outcome measure used or whether the measurement is subjective or objective in nature. Outcomes will be classified using a novel measurement framework (Figure 1). Any outcomes that do not fit within this framework will be classified in an ‘other’ category. Outcomes will be included regardless of the time of measurement.

### Search methods for the identification of studies

We will design and conduct a search strategy using methods recommended by the Institute of Medicine [25], which includes a search of several databases including: PubMed, Scopus, Ovid MEDLINE, Ovid EMBASE, Ovid EBM Reviews CENTRAL, CINAHL, and Ovid PsycInfo. A preliminary search strategy can be found in Additional file 1. The databases will be searched from the time of their inception to the current time. The initial electronic search strategy will be supplemented by screening the reference lists from eligible included studies and through contacting experts in the field to identify any missing, in-progress or unpublished studies. In addition, we will search for reviews on the topic and search through their reference lists to identify any potentially eligible studies that may have been missed through other methods. Finally, clinical trial registries will be searched to identify completed and in-progress studies and, if not identified through other methods, the authors will be contacted for details regarding the study’s status. There will be no language restrictions.

### Selection of studies

We will upload search results into systematic review software (DistillerSR, Ottawa, ON, Canada). In the first round of screening, abstracts and titles will be screened for inclusion. Following abstract screening, eligibility will be assessed through a full-text screening. Prior to both abstract and full-text screening, reviewers will undergo training to ensure a basic understanding of the background of the field and purpose of the review as well as comprehension of the inclusion and exclusion criteria. Eligibility at both levels (abstract and full-text) will be assessed independently and in duplicate. At the title and abstract screening level, both reviewers must be in agreement in order to exclude an article; conflicts will be included. At the level of full-text screening, any disagreements will be resolved by consensus. If consensus cannot be achieved between the two reviewers, a third reviewer will arbitrate.

### Data collection

In each study, we will extract: the outcomes, the time when the outcomes were measured, the estimates of effect for the outcomes and the error associated with those estimates of effect. Furthermore, the instrument(s) used to measure the outcomes in the studies (for example, Control Preferences Scale for the outcome of preference for participation decision making) will also be recorded. Other items that will be extracted include: study year, location (geographic and specialty/primary care) number of participating sites, number of participants in each arm of the trial and average demographics (that is age, sex, race), length of follow-up, losses to follow-up, condition(s) under study, decision being made (that is what decision point is being addressed using the SDM intervention), who developed the intervention (investigator versus pre-developed), description of the intervention and control, theoretical or conceptual underpinning for the study and intervention, type of decision supported (decision to initiate, stop, intensify, or de-intensify treatment), time of intervention delivery (pre-visit/in-visit/post-visit, and so on), mode of intervention delivery (paper/video/web/in-person, and so on), and target of intervention (patient/clinician/both/other).

Prior to data extraction from included studies, a data extraction form will be created and pilot tested by the data extractors on a subset of studies. The extraction form will be changed based on feedback from the extractors to improve usability and ensure completeness. Data extraction will be completed independently and in duplicate. If disagreements arise, they will be resolved by consensus. If a consensus cannot be reached between the two parties, a third reviewer will arbitrate. If data presented in the studies is unclear, missing, or presented in a form that is either unextractable or difficult to reliably extract, the authors of the study will be contacted for clarification. When data extraction is complete, the authors of the studies will be contacted to ensure the accuracy and completeness of the data extraction. In addition, at this time the authors of included studies will be asked if they know of any additional studies, either completed or ongoing, that they believe would be eligible for our review.

Author contact will be initiated by an Email to the corresponding author. If an Email is unavailable, an Internet search will be used to find a current Email address; when Emails are available, first authors of manuscripts will be carbon copied on all Emails to the corresponding author. If Emails for the corresponding author are unavailable, corresponding authors will be contacted by phone. Authors will be given a week to respond to Emails, after which time a follow-up Email will be sent; if no response is received after an additional two weeks, a phone call will be made to try to contact the author. Attempts to reach authors by phone will occur throughout the week for a period of two weeks at which time the author will be classified as uncontactable.

### Risk of bias assessment

We will use the Cochrane Collaboration’s risk of bias tool to evaluate the methodological quality of included studies [26]. The risk of bias in included studies will be assessed in duplicate by reviewers working independently. Any disagreements will be resolved by consensus, if consensus is unable to be achieved, a third reviewer will arbitrate. Items included in the risk of bias assessment will include: randomization, quality of randomization (any important imbalances at baseline), allocation concealment, level(s) of blinding/masking, losses to follow-up, intention to treat analysis, and funding sources. Reviewers will also be given the option to leave full-text comments. Any free-text comments left by the reviewers concerning the risk of bias will also be taken under consideration in determining the risk of bias of the study. This information will be used to inform the interpretation of estimates of effect and may be used as a way to stratify studies in sub-group analyses. Risk of bias (high/low/unclear) will be determined for each study based on the above mentioned factors. The first author along with another reviewer will determine overall risk of bias using objective (number of items positive in the risk of bias assessment) and subjective factors (the importance of the presence or absence of said factors on the studies risk of bias). If there are disagreements between the two reviewers a third reviewer will arbitrate.

### Analysis

We will first summarize and describe the populations, interventions, and outcomes studied. Descriptive statistics will be used as appropriate to compare the characteristics of the studies and narratives will be used as necessary to describe the interventions. Outcomes determined to be similar based on a consensus of reviewers will be pooled. Convenience samples of patients with chronic conditions, clinicians, policy makers, and researchers, will be surveyed and asked to rank the outcomes based on their perceived importance. In addition to pooling the outcomes across all trials, we will stratify the trials based on condition studied. Agreement at the level of screening and for the risk of bias assessment will be measured using the kappa or phi statistics, as appropriate (the latter is appropriate when the distribution of agreement is extreme) [27].

#### Summary measures

We will use Review Manager version 5.2 for statistical analysis [28]. We will use DerSimonian and Laird random-effects models to calculate the relative risk (RR) with 95% confidence intervals [29] for dichotomous variables and weighted mean difference between groups for continuous variables. For outcomes assessed using different measures, we will report the standardized mean difference. A minimum important difference will be defined as 0.5 standard deviations [30]. We will report both random- and fixed-effects models in the case of a discrepancy between them; otherwise, we will report the random-effects model only. Heterogeneity will be assessed using the I^2^ statistic and values above 75% will be considered indicative of high heterogeneity [31].

#### Missing data

If missing data exists within the included trials, we will contact the authors to see if it is obtainable. If the data is unobtainable, we will use the complete case analysis and conduct sensitivity analysis for continuous outcomes and dichotomous outcomes using the methods of Ebrahim *et al*. [32] and Akl *et al*. [33], respectively.

#### Risk of bias across studies

Publication bias will be assessed by plotting the trial’s estimate of effect by the inverse of its standard error using a funnel plot. The plots will be assessed both visually and by using Egger’s test; a significant publication bias will be considered to exist if the *P*-value is < 0.1 [34].

#### Quality of evidence

For each outcome, tables summarizing the quality of the evidence will be generated (that is evidence profiles and summary of findings tables). These evidence summaries will be generated based on guidance formulated by the Grading of Recommendations Assessment, Development, and Evaluation (GRADE) Working Group [35].

#### Additional analyses

Following the primary analyses, several exploratory sub-group analyses will be performed. The analyses will be stratified by: 1) symptomatic conditions versus asymptomatic conditions; 2) target of the intervention (patient/clinician/both/other); 3) time of intervention delivery (pre-consult/in-consult/post-consult); 4) children versus adults; and 5) type of intervention (for example, training versus decision aid). We will also perform a sensitivity analysis including and excluding information gleaned from author contact.

## Discussion

We anticipate that this systematic review will be useful to a variety of stakeholders for several reasons. First, it provides a broad, detailed overview of the field for researchers and will highlight gaps where future research on SDM and its implementation will need to be conducted. Second, it will make available to clinicians knowledge related to the available SDM interventions and their respective efficacies and limitations. Finally, the review could potentially inform policy makers and funding agencies by highlighting the state of the current body of research, the efficacy of different SDM approaches, and where future funding priorities should lie (for example, novel SDM implementation strategy generation versus comparative effectiveness research), which will be informed by an innovative approach to ranking outcomes.

Evidence based medicine is most useful when it considers and fulfills the needs of the end user of that evidence. However, there are often many end users, each with different needs, values, preferences, and contexts. Therefore, to aid in the interpretation and presentation of our review, we will conduct surveys of different end users (patients, clinicians, researchers, and policy makers) to determine which outcomes that have been measured are of most importance to each end user and what, if any, outcomes have not been measured (or have been measured inconsistently) that are important to a specific group of end users. We chose to use surveys over interviews or focus groups because we wanted to generate the desired information as efficiently as possible.

For this review, we propose a novel measurement framework (Figure 1). This framework is tentative and builds off of previous measurement frameworks [36-39]. It is anticipated that this framework will be modified based on results of stakeholder feedback and of this review.

We anticipate that this systematic review will have limitations such as significant heterogeneity and/or imprecision for some of the outcomes and that this will limit our confidence in the estimates of effect. As a result, we may find that for many outcomes, at this time, we are unable to make any conclusions with a high degree of confidence. An additional limitation is that historically, there has been no ‘shared’ definition of what SDM is or what constitutes a good or effective SDM interaction [24]. Thus, despite using a refined definition of SDM there may still be significant conceptual heterogeneity which may affect the interpretation of any results. Therefore, we will conduct sensitivity analysis to explore the effect of this heterogeneity. In addition, we will conduct sensitivity analyses to explore the impact of information gathered from author contact and from different approaches to handling missing data.

We believe this study will be an important contribution to the field as it will highlight the effects interventions aimed at improving the level of SDM have on a variety of outcomes in the setting of chronic disease. We anticipate this information will be useful to a variety of stakeholders and that it will promote discussion both inside and outside the field.

## Abbreviations

SDM: Shared decision making; IPDAS: International Patient Decision Aids Standards; PRISMA: Preferred Reporting Items for Systematic Reviews and Meta-Analyses; RR: Relative risk; GRADE: Grading of recommendations assessment, development, and evaluation.

## Competing interests

The authors declare that they have no competing interests.

## Authors’ contributions

MRG: conception and design, manuscript writing and final approval of the manuscript. ALL: conception and design, critical revision and final approval of the manuscript. JPB: conception and design, critical revision and final approval of the manuscript. AL: conception and design, critical revision and final approval of the manuscript. KRB: conception and design, critical revision and final approval of the manuscript. MAM: conception and design, critical revision and final approval of the manuscript. PJE: conception and design, critical revision and final approval of the manuscript. LJP: conception and design, critical revision and final approval of the manuscript. CLZ: conception and design, critical revision and final approval of the manuscript. GM: conception and design, critical revision and final approval of the manuscript. JJM: conception and design, critical revision and final approval of the manuscript. HM: conception and design, critical revision and final approval of the manuscript. RR: conception and design, critical revision and final approval of the manuscript. RH: conception and design, critical revision and final approval of the manuscript. OLM: conception and design, critical revision and final approval of the manuscript. MHM: conception and design, critical revision and final approval of the manuscript. VMM: conception and design, critical revision and final approval of the manuscript. All authors read and approved the final manuscript.

## Authors’ information

Several of the authors are actively involved in research on shared decision making and have developed several decision aids.

## Supplementary Material

Additional file 1 Search Strategy. Click here for file
