# Supplementary material for: A systematic review of shared decision making interventions in chronic conditions: a review protocol
Source: Syst Rev. 2014 Apr 15;3:38. doi: 10.1186/2046-4053-3-38 (PMC4021633; doi:10.1186/2046-4053-3-38)
Supplement: Additional file 1 — Search Strategy. [file 2046-4053-3-38-S1.docx]

Additional file

Ovid MEDLINE(R) In-Process & Other Non-Indexed Citations and Ovid MEDLINE(R) 1946 to Present

# Searches

1 decision making/ or decision support systems, clinical/ or decision support techniques/ or decisionmaking.mp. or (decision adj (make* or making)).mp. or "clinical decision support".mp. or (decision adj2 aid*1).mp. or "decision support".mp. or decision making, computer assisted/ [mp=title, abstract, original title, name of substance word, subject heading word, keyword heading word, protocol supplementary concept, rare disease supplementary concept, unique identifier]

2 ((support$ or share* or sharing or informed or individual*) adj2 (decid* or decision* or choice)).mp. [mp=title, abstract, original title, name of substance word, subject heading word, keyword heading word, protocol supplementary concept, rare disease supplementary concept, unique identifier]

3 exp professional-patient relations/ or (patient* adj4 (clinician* or provider* or physician* or doctor* or nurse* or professional*)).mp. [mp=title, abstract, original title, name of substance word, subject heading word, keyword heading word, protocol supplementary concept, rare disease supplementary

4 (1 or 2) and 3

5 (1 OR 2) and (patient preference/ or patient participation/)

6 (patient* adj4 (involv* or power or enable* or empower* or engage* or partner* or participat* or collaborat* or consult* or conversation* or prefer* OR EXPECTATION* or realistic OR unrealistic)).mp. [mp=title, abstract, original title, name of substance word, subject heading word, keyword heading word, protocol supplementary concept, rare disease supplementary concept, unique identifier]

Consider (1 or 2) and 6 (?)

7 3 and (patient education as topic/ OR “patient education” or collaborat*.mp. or choice.mp. OR CHOOSE or ask.mp. or questions.mp. or concerns.mp.) [mp=title, abstract, original title, name of substance word, subject heading word, keyword heading word, protocol supplementary concept, rare disease supplementary concept, unique identifier]

8 patient-centered care/ or (patient* adj2 (centered or centred or important or values or goals or perspective or voice or input* or options or sensitive or choice or wishes or perceptions or views)).mp. [mp=title, abstract, original title, name of substance word, subject heading word, keyword heading word, protocol supplementary concept, rare disease supplementary concept, unique identifier]

9 6 and (communication/ or risk*.mp. or clarification.mp. or utility.mp. or benefits.mp. or alternative*.mp. or tradeoff*.mp. or "trade-off*1".mp. or harms.mp.) [mp=title, abstract, original title, name of substance word, subject heading word, keyword heading word, protocol supplementary concept, rare disease supplementary concept, unique identifier]

10 9 and (patient education as topic/ or health knowledge attitudes practice/ or expectations.mp. or autonomy.mp. or coaching/ or proactive.mp. or active.mp. or passive.mp. or prompt*.mp. or style.mp.) [mp=title, abstract, original title, name of substance word, subject heading word, keyword heading word, protocol supplementary concept, rare disease supplementary concept, unique identifier]

11 4 and (7 or 9)

12 8 or 9 or 10

13 (1 or 2) and 12

14 5 or 11 or 13

15 limit 14 to (comparative study or evaluation studies or meta analysis or randomized controlled trial or systematic reviews or validation studies)

16 14 and (quasi* or (pre and post) or preintervention* or baseline or previsit* or postconsult* or (control* and intervention*) OR (BEFORE AND AFTER) (test AND retest)).mp. [mp=title, abstract, original title, name of substance word, subject heading word, keyword heading word, protocol supplementary concept, rare disease supplementary concept, unique identifier]

17 15 or 16

18 14 and (random* or blinded or compar* or meta-analysis or random* or systematic or validat*).mp. [mp=title, abstract, original title, name of substance word, subject heading word, keyword heading word, protocol supplementary concept, rare disease supplementary concept, unique identifier]

19 17 or 18

20 19 AND (chronic* or ex *myocardial ischemia/ or ex *neoplasms/ or ex *heart failure/ or ex *renal insufficiency/ or ex *depressive disorders/ or ex *asthma/ or ex *stroke/ or ex *autism disorder/ or ex *hiv infections/ or ex *diabetes mellitus/ or ex *arthritis/ or ex *osteoporosis/ or ex *hypertension/ or ex *hepatitis/ or ex *multiple sclerosis/ or ex *muscular dystrophies/ )

Or (“chronic illness” OR “chronic disease” OR “chronic condition*” OR “heart failure” OR “cardiac failure” OR “kidney failure” OR “renal failure” OR depression* OR asthma* OR autistic* OR autism* OR “immunodeficien*” OR hiv OR diabetes or diabetic* OR arthritis OR osteoarthrit* OR osteopor* OR “high blood pressure” OR hypertensive OR hypertension OR hepatitis OR “multiple sclerosis” OR “muscular dystroph*” OR “cerebrovascular accident” OR stroke OR cancer OR “coronary artery disease” OR “heart attack” OR “myocardial infarct*” OR dialysis OR hemodialys* OR haemodialys*

(EMBASE) doctor-patient relations

| **Embase** 1988 to 2013 Week 41 | | | |
| --- | --- | --- | --- |
| **#** | **Searches** | **Results** | **Search Type** |
| 1 | decision making/ or decision support systems, clinical/ or decision support techniques/ or decisionmaking.mp. or (decision adj (make* or making)).mp. or "clinical decision support".mp. or (decision adj2 aid*1).mp. or "decision support".mp. or decision making, computer assisted/ [mp=title, abstract, subject headings, heading word, drug trade name, original title, device manufacturer, drug manufacturer, device trade name, keyword] | 252225 | Advanced |
| 2 | ((support$ or share* or sharing or informed) adj2 (decid* or decision* or choice)).mp. [mp=title, abstract, subject headings, heading word, drug trade name, original title, device manufacturer, drug manufacturer, device trade name, keyword] | 30352 | Advanced |
| 3 | exp professional-patient relations/ or (patient* adj4 (clinician* or provider* or physician* or doctor* or nurse* or professional*)).mp. [mp=title, abstract, subject headings, heading word, drug trade name, original title, device manufacturer, drug manufacturer, device trade name, keyword] | 641253 | Advanced |
| 4 | (1 or 2) and 3 | 52020 | Advanced |
| 5 | 4 and (patient preference/ or patient participation/) | 4878 | Advanced |
| 6 | (patient* adj4 (involv* or power or enable* or empower* or engage* or partner* or participat* or collaborat* or consult* or conversation* or prefer*)).mp. [mp=title, abstract, subject headings, heading word, drug trade name, original title, device manufacturer, drug manufacturer, device trade name, keyword] | 177287 | Advanced |
| 7 | 3 and (patient education as topic/ or collaborat*.mp. or choice.mp. or ask.mp. or questions.mp. or concerns.mp.) [mp=title, abstract, subject headings, heading word, drug trade name, original title, device manufacturer, drug manufacturer, device trade name, keyword] | 94456 | Advanced |
| 8 | patient-centered care/ or (patient* adj2 (centered or centred or important or values or goals or perspective or voice or input* or options or sensitive or choice)).mp. [mp=title, abstract, subject headings, heading word, drug trade name, original title, device manufacturer, drug manufacturer, device trade name, keyword] | 161009 | Advanced |
| 9 | 6 and (communication/ or risk*.mp. or clarification.mp. or utility.mp. or benefits.mp. or alternative*.mp. or tradeoff*.mp. or "trade-off*1".mp. or harms.mp.) [mp=title, abstract, subject headings, heading word, drug trade name, original title, device manufacturer, drug manufacturer, device trade name, keyword] | 53387 | Advanced |
| 10 | 9 and (patient education as topic/ or health knowledge attitudes practice/ or expectations.mp. or autonomy.mp. or coaching/ or proactive.mp. or active.mp. or passive.mp. or prompt*.mp. or style.mp.) [mp=title, abstract, subject headings, heading word, drug trade name, original title, device manufacturer, drug manufacturer, device trade name, keyword] | 8689 | Advanced |
| 11 | (1 or 2) and (6 or 8) | 26017 | Advanced |
| 12 | 5 or 10 or 11 | 32853 | Advanced |
| 13 | 12 and chronic*.mp. [mp=title, abstract, subject headings, heading word, drug trade name, original title, device manufacturer, drug manufacturer, device trade name, keyword] | 2933 | Advanced |
| 14 | 13 and randomized controlled trial/ | 129 | Advanced |
| 15 | chronic disease/ | 113626 | Advanced |
| 16 | exp coronary artery disease/ | 182655 | Advanced |
| 17 | exp heart failure/ | 241854 | Advanced |
| 18 | exp *neoplasms/ | 1923826 | Advanced |
| 19 | exp kidney failure/ | 173404 | Advanced |
| 20 | exp asthma/ | 147052 | Advanced |
| 21 | exp autism/ | 31741 | Advanced |
| 22 | exp cerebrovascular accident/ | 59282 | Advanced |
| 23 | exp arthritis/ | 248682 | Advanced |
| 24 | exp *depression/ | 128160 | Advanced |
| 25 | exp *Human immunodeficiency virus infection/ | 212138 | Advanced |
| 26 | exp *diabetes mellitus/ | 273384 | Advanced |
| 27 | exp *hypertension/ | 169070 | Advanced |
| 28 | exp *hepatitis/ | 104517 | Advanced |
| 29 | exp *osteoporosis/ | 39832 | Advanced |
| 30 | exp *multiple sclerosis/ | 42176 | Advanced |
| 31 | exp *hyperlipidemia/ | 33718 | Advanced |
| 32 | or/15-31 | 3888623 | Advanced |
| 33 | 12 and 32 | 9055 | Advanced |
| 34 | 33 and (exp randomized controlled trial/ or meta-analysis/ or systematic review/) | 790 | Advanced |
| 35 | exp *coronary artery disease/ or exp *heart failure/ or exp *neoplasms/ or exp *kidney failure/ or exp *asthma/ or exp *autism/ or exp *cerebrovascular accident/ or exp *arthritis/ or exp *depression/ or exp *Human immunodeficiency virus infection/ or exp *diabetes mellitus/ or exp *hypertension/ or exp *hepatitis/ or exp *osteoporosis/ or exp *multiple sclerosis/ or exp *hyperlipidemia/ | 3417581 | Advanced |
| 36 | 12 and 35 and (quasi* or (pre and post) or preintervention* or baseline or previsit* or postconsult* or (control* and intervention*)).mp. [mp=title, abstract, subject headings, heading word, drug trade name, original title, device manufacturer, drug manufacturer, device trade name, keyword] | 1079 | Advanced |
| 37 | 34 or 36 | 1527 | Advanced |
| 38 | limit 37 to embase | 1227 | Advanced |
| 39 | limit 38 to (book or book series or letter or note or short survey or trade journal) | 12 | Advanced |
| 40 | 38 not 39 | 1215 |  |

CINAHL

S15 S12 OR S14

Limiters - Publication Type: Clinical Trial, Meta Analysis, Meta Synthesis, Randomized Controlled Trial, Research, Systematic Review

S14 S11 AND S13 View Results (463)

S13 TX "shared decision*" View Results (4,439)

S12 S2 AND S11 View Results (243)

S11 S9 AND S10 View Results (10,990)

S10 S7 AND S8 View Results (20,518)

S9 S1 OR S3 OR S4 OR S5 OR S6 View Results (287,699)

S8 Communicat* OR risk* OR clarification OR utility OR benefits OR alternative* OR options OR tradeoff* OR "trade-off*" OR harms OR coach* OR proactive* OR active* OR passive* OR prompt* OR style* OR concordan* OR discord* View Results (571,473)

S7 (MH "Professional-Patient Relations+") OR (MH "Professional-Client Relations") OR (MH "Physician-Patient Relations") OR (MH "Professional-Family Relations") OR (MH "Nurse-Patient Relations") View Results (60,993)

S6 (MH "Patient Centered Care") OR (MH "Patient Care Plans+") View Results (16,417)

S5 patient* AND (involv* OR power OR enable* OR empower* OR engage* OR partner* OR collaborat* OR consult* OR conversation* OR prefer* OR expectation* OR autonomy*OR education* OR realistic* OR unrealistic* OR choice* OR collaborat* OR choose* OR ask* OR concerns OR values OR questions OR voice OR perspective* OR perception* OR important OR input OR wishes OR views) View Results (220,584)

S4 (MH "Consumer Participation") OR "patient participation" View Results (9,905)

S3 (support$ OR share* OR sharing OR informed OR individual* OR personal OR conflict* OR regret* OR adhere* OR complian*) AND (decid* OR decision* OR choice) View Results (36,811)

S2 (MH "Mental Disorders, Chronic") OR (MH "Chronic Disease") View Results (28,844)

S1 (MH "Decision Making+") OR (MH "Decision Making, Patient+") OR (MH "Decision Making, Clinical") OR (MH "Decision-Making Support (Iowa NIC)") OR (MH "Decision Making (Iowa NOC)") View Results (50,708)

| **PsycINFO** 1987 to October Week 5 2013 | | | |
| --- | --- | --- | --- |
| **#** | **Searches** | **Results** | **Search Type** |
| 1 | decision making/ or exp choice behavior/ or exp group decision making/ or exp decision support systems/ or exp risk assessment/ or exp uncertainty/ or exp utility theory/ | 63486 | Advanced |
| 2 | ((shared or aid*1) adj2 decision*).mp. [mp=title, abstract, heading word, table of contents, key concepts, original title, tests & measures] | 2057 | Advanced |
| 3 | 1 or 2 | 64184 | Advanced |
| 4 | ((support$ or share* or sharing or informed or individual* or personal or conflict* or regret* or adhere* or complian*) adj2 (decid* or decision* or choice)).mp. [mp=title, abstract, heading word, table of contents, key concepts, original title, tests & measures] | 11982 | Advanced |
| 5 | exp Medical Patients/ or exp Client Centered Therapy/ or patient centered.mp. or exp Patients/ | 62086 | Advanced |
| 6 | (Communicat* or risk* or clarification or utility or benefits or alternative* or options or tradeoff* or "trade-off*" or harms or coach* or proactive* or active* or passive* or prompt* or style* or concordan* or discord*).mp. [mp=title, abstract, heading word, table of contents, key concepts, original title, tests & measures] | 664235 | Advanced |
| 7 | (4 or 6) and 5 | 18361 | Advanced |
| 8 | chronic.mp. or exp Chronic Illness/ | 84268 | Advanced |
| 9 | 3 and 5 | 1857 | Advanced |
| 10 | (7 or 9) and 8 | 1536 | Advanced |
| 11 | limit 10 to ("0430 followup study" or "0451 prospective study" or "0453 retrospective study" or "0830 systematic review" or 1200 meta analysis or 1600 qualitative study or 1800 quantitative study or "2000 treatment outcome/clinical trial") | 817 | Advanced |
| 12 | limit 11 to all journals | 765 |  |

SCOPUS

(((TITLE-ABS-KEY(((support$ OR share* OR sharing OR informed OR individual* OR personal OR conflict* OR regret* OR adhere* OR complian*) W/2 (decid* OR decision* OR choice)))) OR (TITLE-ABS-KEY("patient participa*" OR "patient satisfaction" OR "patient educat*" OR "patient centered" OR "patient centred" OR "patient knowledge" OR (patient W/3 (relations* OR clinician OR provider OR physician OR doctor OR nurse OR professional)))) OR (TITLE-ABS-KEY(patient* AND (involv* OR power OR enable* OR empower* OR engage* OR partner* OR collaborat* OR consult* OR conversation* OR prefer* OR expectation* OR autonomy*or education* OR realistic* OR unrealistic* OR choice* OR collaborat* OR choose* OR ask* OR concerns OR values OR questions OR voice OR perspective* OR perception* OR important OR input OR wishes OR views))) OR (TITLE-ABS-KEY(communicat* OR risk* OR clarification OR utility OR benefits OR alternative* OR options OR tradeoff* OR "trade-off*" OR harms OR coach* OR proactive* OR active* OR passive* OR prompt*))) AND (TITLE-ABS-KEY(patient W/3 (relations* OR clinician OR provider OR physician OR doctor OR nurse OR professional))) AND (TITLE-ABS-KEY("decision making" OR "decision support" OR decisionmaking OR "decision make*" OR (decision w2 aid*) OR decision* OR decide))) AND ((patient* OR consumer*)) AND (chronic* OR "heart failure" OR "cardiac failure" OR "kidney failure" OR "renal failure" OR depression* OR asthma* OR autistic* OR autism* OR "immunodeficien*" OR hiv OR diabetes OR diabetic* OR arthritis OR osteoarthrit* OR osteopor* OR "high blood pressure" OR hypertensive OR hypertension OR hepatitis OR "multiple sclerosis" OR "muscular dystroph*" OR "cerebrovascular accident" OR stroke OR cancer OR "coronary artery disease" OR "heart attack" OR "myocardial infarct*" OR dialysis OR hemodialys* OR haemodialys* OR bipolar OR schizophren*) AND (trial* OR compar* OR (pre AND post) OR intervention* OR (before AND after) OR (test AND retest) OR qualitative* OR quantitative*) AND (TITLE(patient* OR shared OR consumer*)) AND NOT (PMID(*1 OR *2 OR *3 OR *4 OR *5 OR *6 OR *7 OR *8 OR *9))
